# Variability in Microbial Communities Driven by Particulate Matter on Human Facial Skin

**DOI:** 10.3390/toxics12070497

**Published:** 2024-07-08

**Authors:** Kai Fu, Qixing Zhou, Heli Wang

**Affiliations:** 1 School of Water Resources and Environment, China University of Geosciences (Beijing), Beijing 100083, China; fukai1998@126.com (K.F.);; 2 Key Laboratory of Pollution Processes and Environmental Criteria (Ministry of Education), Tianjin Key Laboratory of Environmental Remediation and Pollution Control, Carbon Neutrality Interdisciplinary Science Centre/College of Environmental Science and Engineering, Nankai University, Tianjin 300350, China

**Keywords:** variability, microbial communities, PM, chemical compositions, health risk

## Abstract

Microbial communities are known to play an important role in maintaining ecological balance and can be used as an indicator for assessing environmental pollution. Numerous studies have revealed that air pollution can alter the structure of microbial communities, which may increase health risks. Nevertheless, the relationships between microbial communities and particulate matter (PM) caused by air pollution in terms of health risk assessment are not well understood. This study aimed to validate the influences of PM chemical compositions on microbial communities and assess the associated health risks. Our results, based on similarity analysis, revealed that the stability structure of the microbial communities had a similarity greater than 73%. In addition, the altered richness and diversity of microbial communities were significantly associated with PM chemical compositions. Volatile organic compounds (VOCs) and polycyclic aromatic hydrocarbons (PAHs) exerted a positive influence on microbial communities in different environmental variables. Additionally, a stronger linear correlation was observed between hydroxyl radicals (·OH) and the richness of microbial communities. All estimated health risks from PM chemical compositions, calculated under different environmental variables, significantly exceeded the acceptable level by a factor of more than 49. Cr and 1,2-Dibromoethane displayed dual adverse effects of non-carcinogenic and carcinogenic risks. Overall, the study provides insights into the fundamental mechanisms of the variability in microbial communities driven by PM, which may support the crucial role of PM chemical compositions in the risk of microorganisms in the atmospheric environment.

## 1. Introduction

Air quality in developing countries, particularly in the Beijing–Tianjin–Hebei Region in China, has become a major concern in recent years, with the connection between air pollution and human health recently obtaining increasing attention based on the findings of several environmental toxicology experiments [1,2]. Human lifestyle, customs, and income levels have led to long-term exposure to outdoor air pollution with higher concentrations of particulate matter (PM), despite conscious efforts to adopt protective measures [3,4]. Previous studies have reported a higher health risk associated with exposure to high concentrations of PM, which increases with unsatisfactory protective measures and weaker individual physiques [5,6]. In addition, given that the face, encompassing the ears, eyes, nose, and mouth, serves as the primary entry point for PM and airborne bacteria into the human body [7,8], it is essential to understand the concentrations of PM, PM chemical compositions, and microbial communities on human facial skin to assess human health risks, regulate air pollution, and improve individual protective measures.

Although every human has their own characterized microbial cloud (microbial communities), a ubiquitous part of the environment, the microbial communities may change as environmental variables such as temperature, humidity, illumination, salinity, pH, etc., are altered [9,10,11]. Changes in microbial communities, including alterations in their concentrations and richness, have been linked to inflammatory diseases, immune responses, and chronic respiratory illnesses, such as asthma and allergic dermatitis [12,13].

Several studies have revealed correlations between microbial communities and PM chemical compositions and reported that these compositions played a significant role in the variability, richness, and diversity of microbial communities [14,15,16]. Laboratory research further demonstrated that heavy metals and polycyclic aromatic hydrocarbons (PAHs) can inhibit or promote the growth of microbial communities, depending on their concentrations and types [17,18]. These studies also suggested that heavy metals, water-soluble anions, PAHs, and volatile organic compounds (VOCs) may enhance the attenuation of bacteria if these toxic pollutants are utilized as carbon or nitrogen sources, or if they induce changes in DNA, enzymes, or albumin [12,19,20]. Mechanistic investigations have revealed the critical role of hydroxyl radicals (·OH) in regulating microbial communities, which may influence their richness and diversity [21]. Moreover, it has been suggested that ·OH radicals may induce substantial changes in microbial communities through metabolic processes, ultimately leading to cellular inactivation [22]. Although numerous studies have been conducted on microbial communities and PM from different separate or mutual perspectives, few studies have assessed the relationship between microbial communities and PM on human facial skin [14,23].

Considering the significant influence of factors such as PM concentrations and temporal variation on the variability in microbial communities driven by PM on human facial skin, it is crucial to examine microbial communities under different PM concentrations and at different times on the human facial skin [24,25]. This would enable the evaluation of environmental risks, determination of associations between PM chemical compositions and microbial communities and development of effective individual protective measures for use in daily life and scientific research.

High-throughput barcoded pyrosequencing of the 16S rRNA gene has been used to detect microbial communities; presently, PM chemical compositions are highly variable in different locations with diverse emission sources through analysis and detection methods that are well-established in the existing literatures [26,27,28,29]. In the present study, canonical correspondence analysis (CCA) combined with orthogonal partial least squares discriminant analysis (OPLS-DA) was used to investigate the association between microbial communities and PM chemical compositions on human facial skin. In addition, linear fitting based on the area of facial skin was used to determine the effects of ·OH by electron paramagnetic resonance (EPR) on the microbial communities.

In this study, we combined PM chemical compositions and microbial communities to describe the compositions and variability in bacterial communities, then determined their correlation on human facial skin at different concentrations of PM and times. Lastly, the health risks associated with exposure to PM were calculated to provide insights for developing novel preventive methods for treating pollutants and formulating measures to minimize the adverse effects caused by PM.

## 2. Materials and Methods

### 2.1. Samples Collection

The samples were collected from college students as fixed sample takers who shared similar characteristics in terms of age (20 to 22 years old), daily routine, diet, and access to air conditioning in Tianjin, China (39°10′ N, 117°10′ E). A total of 480 samples were collected in winter at two distinct times during the day: in the morning after waking up (08:00) and in the evening before sleeping (22:00). These samples were categorized based on two types of air quality conditions: high (>75 μg/m^3^, corresponding to an air pollution index of level three or above in China) and low (≤75 μg/m^3^, corresponding to an air pollution index below level three in China) concentrations of PM_2.5_. To exclude the influence of epidermis and secretions (sweat, fat, etc.) on the results, the students were advised to thoroughly clean their facial skin one hour before morning sampling. Furthermore, samples were collected in the morning and evening periods, ensuring a stable and long exposure time (approximately 15 h per day) and increasing resistance to fluctuating environmental factors such as ozone, UV light, temperature, and humidity. Sterilized medical cotton balls made of absorbent cotton balls (Aladdin, Shanghai, China) were uniformly applied to the surface of the facial skin for collecting PM chemical compositions and microbial communities. In instances free of contamination, a medical cotton ball served as a negative control.

The body surface area was calculated using height and weight parameters based on the Stevenson formula [30,31]. Then, the area of facial skin was calculated using the “Rule of Nines” [32,33]. The specific computational formula is described as follows:

(1) $$S=\frac{0.0061\times Height+0.0128\times Weigh-0.1529}{9}$$

where S represents the area of facial skin (m^2^), height represents the body height (cm), and weight represents the body weight (kg).

A summary of air quality information for the samples was obtained from the China National Environmental Monitoring Centre (CNEMC) and release PM monitoring data on a 1 h cycle. The summary of the sampling collection information is described in Table 1.

### 2.2. 16S rRNA Gene Amplification and Sequencing Processing

Half of each sample was cut into small pieces, and the whole genomic DNA was extracted using an E.Z.N.A.^®^ Soil DNA Kit (Omega Bio-tek, Norcross, GA, USA), following the manufacturer’s protocols. The DNA was extracted in triplicate, and the extracts from the same sample were pooled together and detected using 1% agarose gel electrophoresis. Polymerase chain reaction (PCR, ABI GeneAmp^®^ 9700, Foster City, CA, USA) with the primer sets 515F (5′-barcode-GTGCCAGCMGCCGCGG-3′) and 907R (5′-CCGTCAATTCMTTTRAGTTT-3′) was used to amplify the 16S rRNA gene in the V4–V5 hypervariable regions. Quantitative PCR was performed using the QuantiFluor™-ST (Promega, Fitchburg, WI, USA) blue fluorescence quantitative system after the 16S rRNA gene was amplified. Each process run included a non-template control to assess contamination. The purified amplified samples were sequenced using the Illumina MiSeq platform [34,35]. Operational taxonomic units (OTUs) with a 97% similarity threshold were identified based on classification at seven taxonomic ranks of the Ribosomal Database Project (RDP) classifier. These operations were conducted at Majorbio Biopharm Technology Co., Ltd. (Shanghai, China), and the detailed methods are provided in the Supplementary Materials.

### 2.3. Analysis of Particulate Matter Chemical Compositions

Half of each sample was prepared for PM chemical composition analysis. Briefly, the samples were immersed in 20 mL of ultrapure water for 120 min and then subjected to extraction for 60 min using an ultrasonic bath (KQ-500DE, KunShan Ultrasonic Instruments Co., Ltd., Kunshan, China) with 50 W and a shaker at 150 rpm (HYG-A, TaicangHaochengShiyanYiqiZhizao, Co., Ltd., Taicang, China) for 120 min. The original extracted solutions were obtained after separation by centrifugation (Universal-32R, Heltich, Kirchlengern, Germany) at 1845× *g* for 30 min. The extracted solutions were pooled after two rounds of extraction and filtered through a microporous membrane filter.

Next, a microwave digestion system (MDS-8 Sineo Microwave Chemistry Technology Co., Ltd., Shanghai, China) was used to digest the samples. Additionally, 14 typical heavy metals (Al, As, Ca, Cd, Co, Cr, Cu, Fe, Hg, Mn, Ni, Pb, Sr, and Zn), 4 anions (${SO}_{4}^{2-}$, ${NO}_{3}^{-}$, Cl^−^, and F^−^) of water-soluble ions, 16 priority PAHs, and 35 VOCs were analyzed and quantified. The assessment of heavy metals, water-soluble ions, PAHs, and VOCs was performed as previously reported [14], and the detailed descriptions are presented in the Supplementary Materials (Tables S1 and S2).

### 2.4. Electron Paramagnetic Resonance

The generation of ·OH through a Fenton-type reaction, as a means to assess oxidative stress caused by PM, was performed as previously described [36,37,38]. Briefly, 0.5 mL of the extracted solution was placed in a vial with 25 µL of 500 mM 5,5-dimethyl-1-pyrroline-N-oxide (DMPO, Aladdin, Shanghai, China) as a spin trap; 50 µL of 250 mM H_2_O_2_ was then incubated for 30 min at room temperature in an ultrasonic bath with 50 W after adequately shaking with shaker and vortex mixer. The incubated suspension was transferred into an EPR capillary tube, sealed, and then analyzed using MiniScope-400 EPR spectrometer (Magnettech, Berlin, Germany).

### 2.5. Health Risk Assessment

Health risks from polluted air were considered the chemical compositions of PM with dermal contact route on human facial skin. The dose dermal absorption was estimated by Equation (2) according to the Human Health Evaluation Manual from US EPA [8,39]:

(2) $$Ddermal=\frac{C\times AF\times SA\times ABS\times ED\times EF}{BW\times AT}$$

where C represents the concentrations of PM chemical compositions (μg/m^3^); AF represents the dermal adherence factors (mg/cm^2^); SA represents the total exposed skin surface area (cm^2^); ABS represents the dermal absorption factor; ED represents the exposure duration (years); EF represents the exposure frequency (days/years); BW represents body weight (kg); and AT represents the averaging lifetime (days). The values and units of these parameters are listed in Table 2. The concentrations of PM chemical compositions were determined by an average air flow rate of 8 L/s with ventilation indoors and an average exposure duration of 18 h/day [40].

Non-carcinogenic risk is typically assessed using hazard quotient (HQ). For substances classified as non-carcinogenic, an HQ exceeding 1 indicates a potential for adverse effects. HQ is calculated using Equation (3):

(3) $$HQ=\frac{Ddermal}{RfD}$$

where RfD is chronic reference dose for PM chemical compositions (mg/kg/day) [8,41].

CR is expressed by the product of the dermal absorption dose and the carcinogenic slope factor (SF):

(4) CR=Ddermal×SF

where SF is the carcinogenicity slope factor (mg/kg/day) [41,42].

**Table 2:** Exposure parameter values used in the dose dermal absorption calculations [43,44].

Model Parameters	Abbreviations	Values
Dermal adherence factors	AF	2000 mg/m^2^
Dermal absorption factor	ABS	0.13
Total exposed skin area	SA	m^2^
Exposure duration	ED	25 years
Exposure frequency	EF	313 days/year
Body weight	BW	kg
Averaging time	AT	25,550 days

### 2.6. Statistical Analysis

Singleton sequences were discarded after the process of finding unique sequences was implemented to decrease the redundant computation burden of the analysis procedure. Based on cluster information, the richness estimator and the diversity index were obtained using MOTHUR (http://www.mothur.org/, accessed on 4 March 2023), with a 97% identity threshold. Sorensen community similarity coefficients were calculated to define the community similarity in different environmental variables. All analyses were conducted in three independent experiments with error bars representing standard deviation (mean ± SD). CCA performed in Canoco 5 was used to relate the microbial communities to PM chemical compositions. Collinear variables were removed before the analyses according to Collinearity Diagnostics using the SPSS software (V29), and CCA models were simplified by removing non-significant PM chemical composition variables. A weighted correlation matrix from CCA models was used to relate the effects of PM chemical composition variations that quantify the variation explained and controlled for the effects separately. Further, variable importance in the projection (VIP) and coefficient CS analysis of OPLS-DA were conducted using SIMCA-P+ 14.0 to filter the effects of chemical compositions combined with CCA models. The effects of ·OH on the microbial communities were evaluated using linear fitting based on the facial skin area. A paired-samples t-test was performed to measure the *p* values, and the criteria for significance were set at *p* < 0.05.

## 3. Results

### 3.1. The Variability in Microbial Community Structure

**Figure 1:**
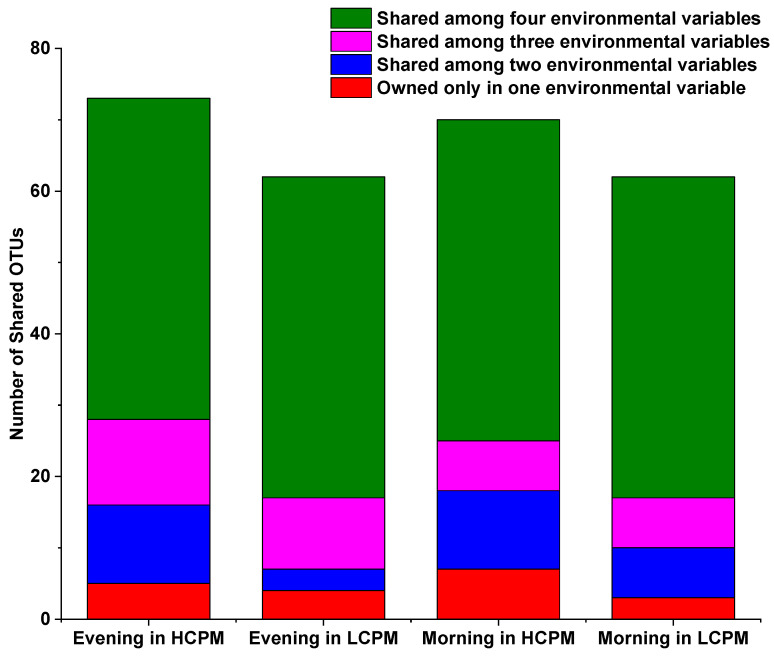
Shared OTUs between different environmental variables during the morning and evening: high concentrations of PM (HCPM) and low concentrations of PM (LCPM).

**Figure 2:**
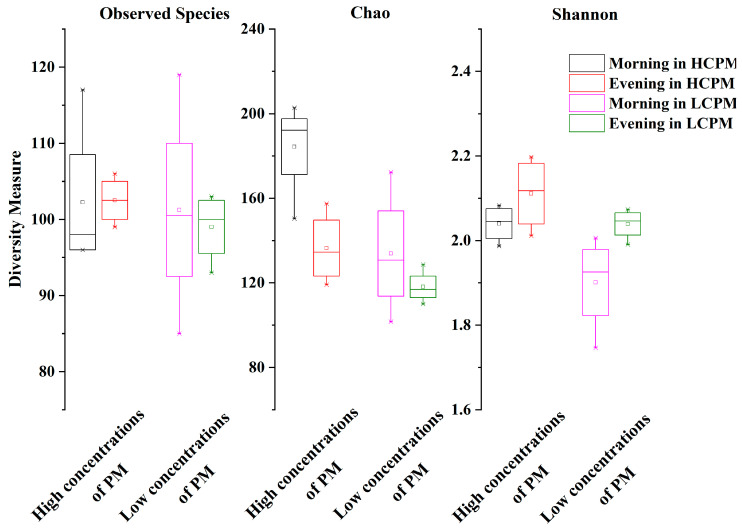
Summary of observed species, richness, and diversity associated with different environmental variables displayed in box plots. Observed species and Chao indexes were plotted to measure richness. Shannon index was used to measure diversity. HCPM: high concentrations of PM; LCPM: low concentrations of PM.

After discarding non-significant sequences, the total number of optimized sequences as reads obtained in each sample ranged from 21,729 to 37,278. Using a 97% similarity threshold, we identified 94–133 OTUs. The paired-sample t-test demonstrated a non-significant difference in OTUs between the samples from high and low PM concentrations (*p* > 0.55). Furthermore, the same result was obtained from the morning and evening samples (*p* > 0.66), indicating no significant difference in the number of OTUs between these time points. However, the number of OTUs increased with increasing concentrations of PM. Interestingly, a similar phenomenon was observed in the morning samples, with higher OTUs observed than in the evening samples. There were ≥45 shared OTUs in all samples. In both morning and evening samples, high PM concentrations were found to be the most abundant shared OTUs, indicating that the microbial communities might have a close association with high PM concentrations (Figure 1). The richness and diversity of microbial communities were investigated to explore the differences in microbial communities among the different samples without taking into account the time required. The findings showed that the Chao indexes were larger in samples collected during high concentrations of PM than those collected during low concentrations of PM (*p* = 0.02), indicating that the richness of microorganisms was higher in high concentrations of PM (Figure 2). Additionally, there were significant differences (*p* = 0.03) in the Shannon index between the two concentrations of PM, with higher diversity observed in higher concentrations of PM. Similar results were found between the morning and evening samples, with Chao and Shannon indexes indicating that microbial communities had higher richness and lower diversity in the morning samples (Figure 2). Both the Chao (*p* = 0.05) and Shannon (*p* = 0.04) indexes were significantly different between the morning and evening samples. The observed species were more abundant in higher concentrations of PM and in the morning, although the differences were not significant (*p* > 0.60) (Figure 2). Collectively, these findings suggest that both concentrations of PM and temporal variations had an important influence on microbial communities on human facial skin.

**Figure 3:**
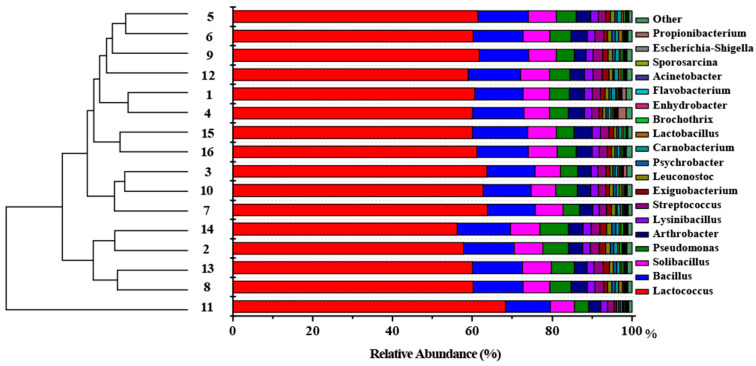
Taxonomic composition and similarity of all samples at the genus level. The relative abundance of the genera is listed on the right, and the remaining genera in each library are represented by “others”. The similarity of relative abundance among all samples is displayed on the left.

The taxonomic classification of bacteria based on the taxonomic rank of the RDP classifier was implemented for characterizing microbial community structure. The microbial community structure showed a low variability in all samples (Figure 3). *Lactobacillales*, *Bacillales*, *Solibacillus*, and *Pseudomonadales* were the most abundant bacterial taxa, comprising approximately 83% of all communities. To further explore the variability in the microbial community in different concentrations of PM and at different times, the modified Sorensen community similarity coefficients were calculated using the following calculation formula assisting similarity [45,46]:

(5) $$S=\frac{2c}{a+b}$$

where S represents the community similarity coefficient; a represents the species in the sample (community) A; b represents the species in the sample (community) B; and c represents the shared species in samples (communities) A and B.

The similarity coefficient in samples collected during high concentrations of PM was 79.61%, which is higher than that in samples collected during low concentrations of PM (73.61%). Similarly, the similarity coefficients in morning and evening samples were 76.66% and 78.20%, respectively, and the Sorensen community similarity coefficients calculated above were in accordance with shared OTUs, as shown in Figure 1. Further, we also observed that the microbial community structure tended to differ more between different concentrations of PM, although the difference was non-significant.

### 3.2. Relationships between Microbial Communities and Chemical Compositions

The relationships between microbial communities and chemical compositions were analyzed by quantifying 14 typical heavy metals, 4 water-soluble anions, 16 priority PAHs, and 35 VOCs. The results showed that the concentrations of all analyzed chemical compositions analyzed ranged from 74.40 to 254.38 mg/m^2^ in the samples collected under different sampling conditions (Table S3). Both richness and diversity indexes were found to decrease with decreasing concentrations of PM, and the morning samples had higher richness and lower diversity (Table S3 and Figure 2), indicating that the structure of microbial communities varied according to the concentrations of PM chemical compositions collected from human facial skin. Eleven effective variables in PM, specifically Ca and Cr of heavy metals, Acenaphthene (Acen), Benzo[k]fluoranthene (BkF), and Indeno[1,2,3-cd]pyrene (InP) of PAHs, Toluene, 1,2-Dibromoethane, Chlorobenzene, p-Xylene, and 1,3,5-Trimethylbenzene of VOCs, and ${NO}_{3}^{-}$ of water-soluble ions were optimized from all the chemical compositions. After eliminating the collinear variables that showed a strong correlation with other chemical compositions using Collinearity Diagnostics (Tolerance ≤ 0.1 or VIF ≥ 10), the concentrations of the remaining variables, considered effective variables, were analyzed. These concentrations ranged from 0.71 to 2.88 mg/m^2^, constituting between 0.44% and 1.87% of all PM chemical compositions (Table S3), thereby revealing the critical roles of the effective variables played on microbial communities despite the small percentages.

**Figure 4:**
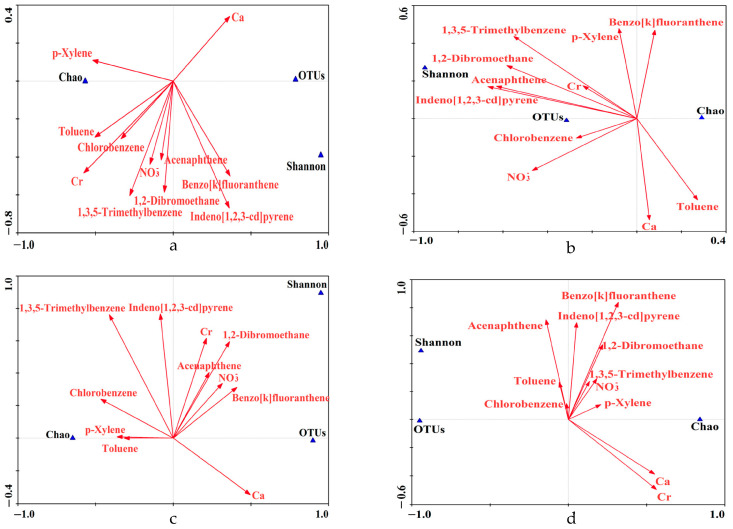
The relation between microbial communities diversity and PM chemical compositions in different environmental variables based on CCA. (**a**) High concentrations of PM; (**b**) low concentrations of PM; (**c**) in the morning; (**d**) in the evening.

**Figure 5:**
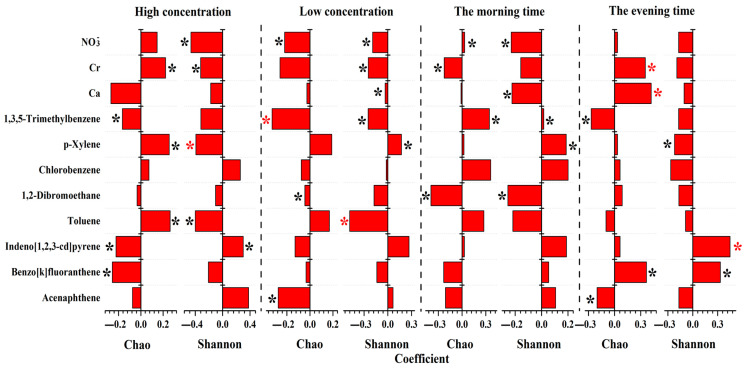
The effects of PM chemical compositions on microbial richness/diversity based on OPLS-DA. Chao index indicates microbial richness, and Shannon index indicates microbial diversity. The black and red asterisks indicate the variable importance in the projection (VIP) values exceeding 1 and 1.5, respectively.

CCA and OPLS-DA were conducted on 11 effective variables and 3 indicators (number of all OTUs, Chao index, and Shannon index) of microbial communities to investigate the potential correlation between microbial communities and PM chemical compositions on human facial skin. The results of the CCA and OPLS-DA provided insights into the relationship between the microbial communities and PM chemical compositions (Figure 4 and Figure 5). The first two axes of the CCA had inertia of 1.000, and the correlations between the three indicators and effective variables were 99.3% and 100%, respectively, indicating that the CCA analysis accurately described the relationship between the three indicators and effective variables and that the previous application of Collinearity Diagnostics was successful. The weighted correlation matrix of the CCA showed a strong relationship between the Ace index and OTUs, which was consistent with the definition of the Ace index. Therefore, in the following analysis, only the impacts of PM on the Ace index and Shannon index were analyzed.

As shown in Figure 4 and Figure 5, it can be observed that Chlorobenzene had a minimal impact and contribution on microbial communities across different concentrations of PM and times. On the other hand, p-Xylene showed a positive correlation with the Chao index of microbial communities in higher concentrations of PM. However, InP and BkF displayed a negative correlation, indicating a substantial impact of these chemical compositions with VIP > 1.14. In addition, InP demonstrated a positive correlation with the Shannon index of microbial communities (VIP > 1.06), while p-Xylene was found to have a negative impact, with a VIP > 1.02. In cases of lower concentrations of PM, we observed a positive correlation between Toluene and the richness of microbial communities. Nevertheless, ${NO}_{3}^{-}$, 1,2-Dibromoethane, Acen, and especially 1,3,5-Trimethylbenzene (VIP > 1.93), were found to have a negative impact on microbial communities. Further, a positive correlation between p-Xylene and the diversity of microbial communities (VIP > 1.14) was observed, while Ca, especially Toluene (VIP > 1.78), demonstrated a negative correlation with the Shannon index.

Our results also showed that 1,3,5-Trimethylbenzene had a positive influence on the richness of microbial communities in the morning samples (VIP > 1.19), while Cr exerted a negative distinctive impact. Although Acen and BkF, but not Toluene, were found to have a positive impact on the Shannon index, the VIP analysis showed that the impact was not significant. In the evening samples, CCA indicated a positive correlation between the richness of microbial communities and BkF, Ca, and Cr, with VIP > 1.20 except for Toluene and Acen. Further, InP and BkF had a positive impact (VIP > 1.38), while Ca and Cr had a negative and non-significant impact (VIP < 0.88) on the diversity of microbial communities.

### 3.3. Reactive Species Influence Microbial Communities

**Figure 6:**
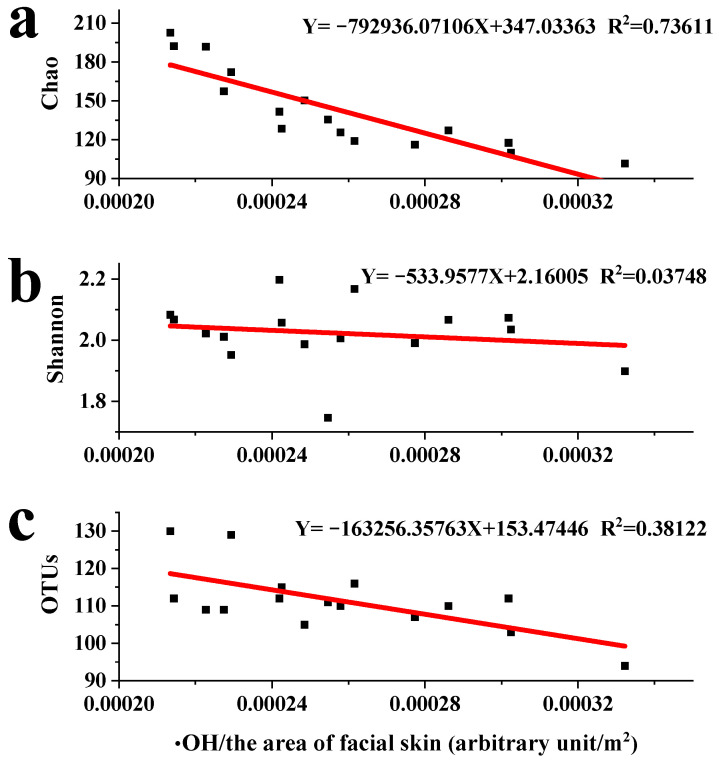
The effects of ·OH on microbial communities. (**a**–**c**) The potential effects of ·OH on richness and diversity indexes Chao, Shannon, and OTUs of microbial communities, respectively.

To further explore the effects of PM chemical compositions on microbial communities, the ·OH of the most important reactive species was analyzed by EPR. The results showed that the ·OH concentrations were significantly lower in low PM concentrations compared to those in high PM concentrations (*p* = 0.04). In addition, the ·OH concentrations increased with time from morning to evening. The correlations between ·OH and microbial communities, in terms of Chao, Shannon, and OTUs, were calculated using the arbitrary unit of ·OH in the unit area of the facial skin (Figure 6). The Chao index was found to decrease as the ·OH concentration increased per unit area of facial skin; the Pearson’s correlation coefficients were higher than 0.70 using linearity fitting, indicating that the richness of the microbial community was potentially suppressed to some extent by the ·OH concentration (Figure 6a). However, the fitting of ·OH and Shannon index was not significant (R^2^ < 0.04), indicating that the effects of ·OH on the diversity of microbial communities were not significant, which conflicted with the Chao index (Figure 6b). A similar phenomenon was observed for ·OH and OTUs (R^2^ < 0.40), thereby supporting the results for the effects of ·OH on microbial communities (Figure 6c).

### 3.4. Health Risks from PM

In this study, human health risk exposure to PM chemical compositions via dermal adsorption was calculated. Cr exhibited the highest non-carcinogenic risk with an HQ exceeding 2.63, which is significantly greater than other PM chemical compositions. Conversely, Toluene had the lowest HQ value (1.22 × 10^−4^). The HQ of the top three risk contributors (Cr, 1,2-Dibromoethane, and 1,3,5-Trimethylbenzene) decreased in this order for both high and low PM concentrations. Interestingly, this trend was also observed in both morning and evening samples, suggesting that Cr, 1,2-Dibromoethane and 1,3,5-Trimethylbenzene pose a higher non-carcinogenic risk in different environmental variables. The carcinogenic risk assessment of PM chemical compositions showed that Cr has the highest risk associated with dermal contact, with a mean value of 29.08 × 10^−4^, even the minimum value (5.98 × 10^−4^) exceeds the risk that is unacceptable according to the EPA. The carcinogenic risks of 1,2-Dibromoethane were also confirmed in this study with the mean value of CR was 5.67 × 10^−4^ (ranging from 4.74 × 10^−5^ to 11.61 × 10^−4^).

## 4. Discussion

It is well established that microbial communities are known to shift to some extent in response to changes in environmental variables [47,48]. Moreover, these shifts in microbial communities may often correspond to competitive dynamics that occur when certain bacteria are exposed to ideal (or suboptimal) conditions related to food availability, temperature, humidity, salinity, pH, and other environmental factors [49,50]. This study successfully determined the variability in microbial communities driven by PM and their correlation on human facial skin in different environmental variables based on the characterizations of PM chemical compositions, which was supported by a shift in microbial community structure and PM chemical compositions from the laboratory experiments described above.

The study revealed a higher number of species in high concentrations of PM and during the morning, which was consistent with the Chao index (Figure 2). This phenomenon can be explained by three possible factors. First, it could be possible that some microbes can more easily utilize carbon and energy sources present in high concentrations of PM [14,51]. Second, microbial communities may tend to seek refuge from adverse nighttime atmospheric conditions, such as UV exposure and rapid temperature fluctuations, which could occur while people are sleeping [14]. Third, microbes on human facial skin may propagate more quickly during the night, as they may utilize secretions from the skin that accumulate during sleep [52]. Further, we found that microbial communities closely interacted in high concentrations of PM, as evidenced by the shared OTUs shown in Figure 1, suggesting that the chemical compositions of PM, as well as environmental variables such as airflow, temperature, the availability of suitable food sources, and the presence of human secretions played similar roles in shaping the microbial communities [52]. The larger diversity of microbial communities in high concentrations of PM (Figure 2) indicated that higher concentrations of PM might influence the composition of microbial communities, similar to that documented in a previous study [53]. Additionally, the study revealed that microbial communities exhibited higher richness and lower diversity in the morning compared to the evening (Figure 2), suggesting that microbes thrived more actively during nighttime in milder environmental conditions, which may provide protection against harsh atmospheric factors and foster heterotrophic activity, aligning with findings from previous studies [26,54]. However, during the daytime, factors such as increased airflow, crowd flow, and microbial immigration via airborne dust may contribute to an increase in the diversity of microbial communities due to the introduction of other bacteria [55].

In this study, the analysis of human facial skin samples revealed a predominance of Gram-positive microbes, including a subset of microbial genera (*Lactococcus*, *Bacillus,* and *Arthrobacter*) known to be associated with acid production, which may impact human health and induce skin and intestinal diseases [56,57]. Further, the structural cell wall compositions and thickness of Gram-positive microbes allow them to remain viable for longer durations in the air, which may cause more damage to humans [58]. Despite the observed changes in microbial richness and diversity with significant shifts (*p* < 0.05) from human samples in different environmental variables (Figure 2), the overall stability of the microbial community structure remained relatively unchanged. In particular, dominant bacterial genera such as *Lactococcus* and *Bacillus* did not exhibit significant changes in abundance over time. This finding indicates that microbial communities on human facial skin exhibit stress resistance, enabling them to withstand environmental attenuation, PM-induced toxicity, and natural enemies [59]. Overall, the stability of these microbial communities could be influenced by PM and change substantially over time, characterized by alterations in their richness and diversity, as shown by the CCA and OPLS-DA results.

The concentrations of PM chemical compositions, including heavy metals, water-soluble ions, PAHs, and VOCs, were measured in different environmental samples (Table S3). The results showed that in environments with high concentrations of PM and appropriate temperatures, degrading bacteria could be incubated to degrade p-Xylene for ecological balance, thereby contributing to an increase in the enrichment of microbial communities [60]. In low concentrations of PM, with the presence of Toluene and appropriate environmental conditions, certain microbial species may actively metabolize this compound [61]. Moreover, bacteria can use toluene sulfonate, which is produced from Toluene and sulfate, as the sole source of sulfur, carbon, and electron acceptors to enhance the richness of microbial communities [62]. However, Toluene-degrading bacteria such as *Pseudomonas* are dominant in high-Toluene environments and may reduce microbial diversity [63].

This study showed that the richness of microbial communities correlated with Cr, but opposite results were observed in regard to diversity in the evening. In addition, Cr was found to have an important effect on the assembly of microbial communities, whereas microbial communities could be negatively affected by Cr stress over time by inhibiting most metabolic pathways and functional genes [64,65]. In the present study, the richness of microbial communities increased significantly with Ca, while restricted diversity was observed in the evening, reflecting the dual character of the influence of Ca [66,67].$\;{NO}_{3}^{-}$ played a negative effect on the growth and reproduction of some bacteria, especially basophilic bacteria, similar to that reported in a previous study [68]. The impact may be even more severe in acidic aerosols, where heavy metals and organic matter can recombine and further alter bacterial enzymatic activity and metabolism. Despite this, certain bacteria may still be able to acquire nitrogen nutrition from ${NO}_{3}^{-}$ to increase the richness of microbial communities [69].

Our study analysis also revealed that InP positively impacted the diversity of microbial communities across varying concentrations of PM. Additionally, it has been shown that, under appropriate conditions, the introduction of certain bacteria to soil can lead to the depletion of InP, which is crucial in determining the effectiveness of a remedial strategy [70]. As the concentrations of PAHs, particularly high-molecular-weight PAHs such as InP, increase, specific degradation bacteria may emerge to effectively reduce the levels of these pollutants, which can ultimately lead to an increase in the diversity of microbial communities [70]. Additionally, laboratory studies have also shown that BkF is highly effective in enhancing enzyme activity, specifically in the degradation of high-molecular-weight PAHs, thereby significantly increasing the richness and diversity of microbial communities in evening samples [71].

Reactive oxygen species (ROS), highly correlated with organic compounds and transition metals, are thought to cause oxidative stress as the main mechanism of PM toxicity in aerosols [72]. ·OH, an important ROS typically present in the open air, is known to be highly reactive and can cause significant environmental health risks through oxidative damage [73]. The concentrations of ·OH were found to significantly increase with increased PM concentrations (*p* = 0.04), possibly due to the increase in the effectiveness of organic compounds and transition metals with changing environmental conditions. The concentrations of ·OH from human facial skin tended to increase after exposure to air in one day, but the difference was not significant (*p* > 0.2). Linear fitting of ·OH and microbial community indexes exhibited preeminent fitting in terms of the Chao index, indicating that ·OH might play an important role in the enrichment of microbial communities (Figure 6a), possibly due to ·OH as the production from PM-inhibited bacteria growth and the appearance of new bacteria by oxidative stress [74]. However, the role of ·OH on microbial community diversity was not obvious. Our results showed that heavy metals and organic compounds might have more effect than ·OH on microbial community diversity, and recombination action played a more important role on the microbial communities.

HQ, representing non-carcinogenic risk, revealed that Cr, 1,2-Dibromoethane, and 1,3,5-Trimethylbenzene were the top three PM chemical compositions with highest non-carcinogenic risks, which indicated that these PM chemical compositions might pose a non-carcinogenic risk to humans. The US EPA recommends an acceptable level of 1 × 10^−6^ as the criterion of health risk identification, whereby values exceeding this threshold indicate a substantial risk [8,39]. However, the carcinogenic risks calculated in our study ranged from 4.74 × 10^−5^ to 66.32 × 10^−4^ in different environmental variables, significantly exceeding the acceptable level stated by the US EPA, indicating that the carcinogenic risk from PM (especially Cr and 1,2-Dibromoethane) for residents in the exposed population could be of great concern. The findings of this study suggest that the dermal adsorption of PM chemical compositions poses potential non-carcinogenic and carcinogenic risk that should not be ignored. Furthermore, the health risks show a significant increase when considering the ingestion and inhalation of PM through the mouth and nose. Therefore, greater emphasis is recommended on health risks associated with long-term exposure to PM chemical compositions, and the potential harm to human health should not be overlooked, especially in places with significant air pollution. Thus, it is strongly recommended that air pollution be controlled, and measures for health protection be implemented for residents in affected regions.

## 5. Conclusions

In this study, we analyzed microbial communities in different concentrations of PM and at different times on facial skin. Through high-throughput techniques, we observed a stable community structure and regular richness and diversity of microbial communities, which were confirmed to be impacted by the chemical compositions of PM. Through Collinearity Diagnostics, we identified 11 effective variables from all PM chemical compositions, and the results suggest that Chlorobenzene had a minimal impact and contribution on microbial communities. The richness of microbial communities was positively impacted by VOCs in different concentrations of PM, whereas the diversity of microbial communities was positively impacted by PAHs at different times. Moreover, there was an association between heavy metals, such as Ca and Cr, and altered richness and diversity of microbial community in evening samples. Reactive species analysis revealed that ·OH and richness of microbial communities had a better linear relationship, although this phenomenon was not observed in terms of diversity.

In conclusion, the variability in microbial communities driven by PM and the stability structure possessed by microbial communities were assessed in this study, which showed that *Lactobacillales*, *Bacillales*, *Solibacillus,* and *Pseudomonadales* were the most abundant bacteria. All estimated health risks from PM chemical compositions, calculated under different environmental variables, significantly exceeded the acceptable level set by the US EPA. Crucially, Cr and 1,2-Dibromoethane were identified as posing dual threats, exhibiting both non-carcinogenic and carcinogenic risks.

Overall, this present study provides in-depth insights into the fundamental mechanisms of the variability in microbial communities driven by PM. Importantly, the findings reveal the potential health risks associated with PM chemical compositions on human facial skin, emphasizing the urgent need for controlling major pollutants and for individuals to take protective measures such as wearing masks or maintaining facial skin cleansing when exposed to open polluted air. These findings offer valuable information for the development of strategies aimed at mitigating the adverse effects of PM on human health.

## Figures and Tables

**Table 1:** Summary of the sampling collection information.

Samples Collection	Concentrations of PM_2.5_/μg/m^3^	Concentrations of PM_10_/μg/m^3^
High concentrations of PM	139	183
	207	290
	166	202
	160	196
Low concentrations of PM	39	54
	45	79
	27	50
	49	85

## Data Availability

The raw data supporting the conclusions of this article will be made available by the authors on request.

## Supplementary Materials

Analytical methods for PM properties and microbial communities; Tables of 16 PAHs and 35 VOCs; Concentrations of the main chemical compositions. The following Supplementary Materials can be downloaded at: https://www.mdpi.com/article/10.3390/toxics12070497/s1, Table S1: Summary of 16 PAHs priority pollutant information; Table S2: Summary of 35 VOCs information; Table S3: Concentrations of the main chemical compositions in human facial skin (mg/m^2^). References [75,76,77,78,79] are cited in the Supplementary Materials.

## Abbreviations

PM	particulate matter
US EPA	US Environmental Protection Agency
PAHs	polycyclic aromatic hydrocarbons
VOCs	volatile organic compounds
CCA	canonical correspondence analysis
OPLS-DA	orthogonal partial least squares discriminant analysis
·OH	hydroxyl radicals
EPR	electron paramagnetic resonance
CNEMC	China National Environmental Monitoring Centre
PCR	polymerase chain reaction
Operational taxonomic units	OTUs
RDP	Ribosomal Database Project
DMPO	5,5-dimethyl-1-pyrroline-N-oxide
HQ	hazard quotient
CR	carcinogenic risk
SF	slope factor
AF	dermal adherence factors
ABS	dermal absorption factor
SA	skin area
ED	exposure duration
EF	exposure frequency
BW	body weight
AT	averaging time
NaP	naphthalene
Acy	acenaphthylene
Acen	acenaphthene
Fl	fluorene
Phe	phenanthrene
Ant	anthracene
Flu	fluoranthene
Pyr	pyrene
BaA	benzo[a]anthracene
Chr	chrysene
BbF	benzo[b]fluoranthene
BkF	benzo[k]fluoranthene
BaP	benzo[a]pyrene
InP	indeno[1,2,3-cd]pyrene
DbA	dibenzo[a,h]anthracene
BgP	benzo[g,h,i]perylene
VIP	variable importance
