# Supplementary material for: Variability in Microbial Communities Driven by Particulate Matter on Human Facial Skin
Source: Toxics. 2024 Jul 8;12(7):497. doi: 10.3390/toxics12070497 (PMC11280976; doi:10.3390/toxics12070497)
Supplement: Supplementary file 1 [file toxics-12-00497-s001.zip › toxics-3038419-supplementary.pdf]

toxics

Variability in Microbial Communities Driven by Particulate Matter on Human Facial Skin

Kai Fu [1,2], Qixing Zhou [2,*] and Heli Wang [1]

1 School of Water Resources and Environment, China University of Geosciences (Beijing), Beijing 100083, China.
2 Key Laboratory of Pollution Processes and Environmental Criteria (Ministry of Education), Tianjin Key Laboratory of Environmental Remediation and Pollution Control, Carbon Neutrality Interdisciplinary Science Centre/College of Environmental Science and Engineering, Nankai University, Tianjin 300350, China.
* Correspondence: author: zhouqx@nankai.edu.cn, Tel: 86-22-23507800, Fax: 86-22-23507800

Materials and methods

16. S rRNA gene amplification and sequencing processing

PCR conditions were as follows: (a) initial denaturation at 95 °C for 3 min; (b) 27 cycles at 95 °C for 30 s, 55 °C for 30 s, and 72 °C for 45 s; (c) a final extension at 72 °C for 10 min. The PCR amplification reactions were performed in a TransGen AP221-02. TransStartFastpfu DNA Polymerase was used for PCR reactions (20 μL total volume) in the following reaction mixture: 4 μL of 5×FastPfu Buffer, 2 μL of 2.5 mM dNTPs, 0.8 μL of Forward Primer (5 μM), 0.8 μL of Reverse Primer (5 μM), 0.4 μL of FastPfu Polymerase, 10 ng of template DNA, and 12 μL of ddH2O. The extracted amplified samples were washed with Tris-HCl and purified using the PCR clean-up system (Promega) and AxyPrep DNA Gel Extraction Kit (Axygen Biosciences, California, USA) according to the manufacturer's instructions. The sequencing data were optimized based on the following criteria: (i) Poor-quality bases of 250 bp reads were truncated if the average quality score was <20 across a 50 bp sliding window, discarding those shorter than 50 bp back-end reads. (ii) Paired reads were merged according to the relation between PE reads and only sequences that overlapped for more than 10 bp were assembled. (iii) The maximum mismatch ratio for the overlap region of merged sequence was 0.2 to screen for unqualified sequences. (iv) Sequences were distinguished according to the barcode on the end of reads and by the amplification primer: barcode mismatch accepted at 0 and maximum mismatch accepted at 2.

Analysis of heavy metals

The samples were placed in a Teflon vessel and initially extracted with concentrated acid solutions (2.5 mL HNO_3, 99.0%; 1.0 mL H_2O_2, 30%; 1.5 mL HF) through temperature programming: (i) 120 °C for 20 min; (ii) 190 °C for 1 h. After cooling to room temperature, the sample in the Teflon vessel was boiled to dryness on a hot plate (190 °C). The sample in the Teflon vessel was boiled to dryness after cooling to room temperature, then diluted with ultrapure water (18MΩ/cm, Milli-Q) at room temperature. The solution was transferred to a clean centrifuge tube after filtration through a microporous membrane filter (pore size, 0.22 μm; diameter, 25 mm, Beijing Dingguo Changsheng Biotechnology Co., Ltd, Beijing, China).

Analysis of water-soluble ions

The samples were dipped into 10 mL ultrapure water for 30 min and then extracted for 45 min using an ultrasonic bath with 50 W (KQ-500DE, KunShan Ultrasonic Instruments Co., Ltd, Kunshan, China) and a shaker at 150 rpm (HYG-A, TaicangHaocheng-Shiyanyiqizhizao, Co., Ltd, Taicang, China). The extracted solution was pooled after two rounds of extraction. The sample fragments of medical cotton balls were separated from extracted solution by centrifugation (Universal-32R, Heltich, Germany) at 1,845 g for 5 min.

Analysis of PAHs and VOCs

Briefly, the samples were placed in a 100 mL glass bottle, mixed with normal hexane/dichloromethane (1:1, v/v), and extracted in an ultrasonic bath for a total of 45 (315) min at 50 W. Each sample was extracted thrice for the same process and the extracted solutions were pooled in a clean glass bottle. The extracts were filtered and concentrated to 5 mL in a rotatory evaporator (Heizbad HB digit, Heidolph, Germany) and then purified by solid-phase extraction (CNWBOND Si SPE Cartridge, Anpel, Shanghai, China). The final volume of the extracted solution was brought to 1 mL using normal hexane after drying by nitrogen and then stored in vials at -40 °C until analysis.

Table S1. Summary of 16 PAHs priority pollutant information.

Serial number	Name	Abbreviation	CAS No.	Molecular weight
1	Naphthalene	NaP	91-20-3	128.18
2	Acenaphthylene	Acy	208-96-8	152.20
3	Acenaphthene	Acen	83-32-9	154.20
4	Fluorene	Fl	86-73-7	166.23
5	Phenanthrene	Phe	85-01-8	178.24
6	Anthracene	Ant	120-12-7	178.24
7	Fluoranthene	Flu	206-44-0	202.26
8	Pyrene	Pyr	129-00-0	202.26
9	Benzo[a]anthracene	BaA	56-55-3	228.30
10	Chrysene	Chr	218-01-9	228.30
11	Benzo[b]fluoranthene	BbF	205-99-2	252.32
12	Benzo[k]fluoranthene	BkF	207-08-9	252.32
13	Benzo[a]pyrene	BaP	50-32-8	252.32
14	Indeno[1,2,3-cd]pyrene	InP	193-39-5	276.34
15	Dibenzo[a,h]anthracene	DbA	53-70-3	278.35
16	Benzo[g,h,i]perylene	BgP	191-24-2	276.34

Table S2. Summary of 35 VOCs information.

Serial number	Name	CAS No.	Molecular Formula	Molecular weight
1	1,1-Dichloroethene	75-35-4	$C_2H_2Cl_2$	96.94
2	1,1,2-Trichloro-1,2,2-trifluormethane	76-13-1	$C_2Cl_3F_3$	187.38
3	Allyl chloride	107-05-1	C_3H_5Cl	76.52
4	Methylene chloride	75-09-2	CH_2Cl_2	84.93
5	1,1-Dichloroethane	75-34-3	$C_2H_4Cl_2$	98.96
6	cis-1,2-Dichloroethene	156-59-2	$C_2H_2Cl_2$	96.94
7	Trichloromethane	67-66-3	$CHCl_3$	119.38
8	1,1,1-Trichloroethane	71-55-6	$C_2H_3Cl_3$	133.40
9	Carbon tetrachloride	56-23-5	CCl_4	153.82
10	1,2-Dichloroethane	107-06-2	$C_2H_4Cl_2$	98.96
11	Benzene	71-43-2	C_6H_6	78.11
12	Trichloroethylene	79-01-6	C_2HCl_3	131.39
13	1,2-Dichloropropane	78-87-5	$C_3H_6Cl_2$	112.98
14	cis-1,3-Dichloropropene	10061-01-5	$C_3H_4Cl_2$	110.97
15	Toluene	108-88-3	C_7H_8	92.14
16	trans-1,3-Dichloropropene	10061-02-6	$C_3H_4Cl_2$	110.97
17	1,1,2-Trichloroethane	79-00-5	$C_2H_3Cl_3$	133.40
18	Tetrachloroethylene	127-18-4	C_2Cl_4	165.83
19	1,2-Dibromoethane	106-93-4	$C_2H_4Br_2$	187.86
20	Chlorobenzene	108-90-7	C_6H_5Cl	112.56
21	Ethylbenzene	100-41-4	C_8H_{10}	106.16
22	m-Xylene	108-38-3	C_8H_{10}	106.16
23	p-Xylene	106-42-3	C_8H_{10}	106.16
24	o-Xylene	95-47-6	C_8H_{10}	106.16
25	Styrene	100-42-5	C_8H_8	104.15

26	1,1,2,2-Tetrachloroethane	630-20-6	$C_2H_2Cl_4$	167.86
27	4-Ethyltoluene	622-96-8	C_9H_{12}	120.19
28	1,3,5-Trimethylbenzene	108-67-8	C_9H_{12}	120.19
29	1,2,4-Trimethylbenzene	95-63-6	C_9H_{12}	120.19
30	1,3-Dichlorobenzene	541-73-1	$C_6H_4Cl_2$	147.00
31	1,4-Dichlorobenzene	106-46-7	$C_6H_4Cl_2$	147.00
32	Benzyl chloride	100-44-7	C_7H_7Cl	126.58
33	1,2-Dichlorobenzene	95-50-1	$C_6H_4Cl_2$	147.00
34	1,2,4-Trichlorobenzene	120-82-1	$C_6H_3Cl_3$	181.45
35	Hexachlorobutadiene	87-68-3	C_4Cl_6	260.76

Analysis of particulate matter chemical compositions

A total of 14 typical heavy metals (Al, As, Ca, Cd, Co, Cr, Cu, Fe, Hg, Mn, Ni, Pb, Sr, and Zn) were analyzed and the measuring conditions were assessed by inductively coupled plasma optical emission spectroscopy (VISTA-MPX, Varian, USA). Four anions (SO_4^{2-}, NO_3^-, Cl^-, and F^-) were analyzed by an ion chromatography (ICS-5000, Thermo Fisher Scientific, USA) with a Dionex AS16 Column. Further, 16 priority PAHs and 35 VOCs were quantified by gas chromatography–mass spectrometry (GC-MS, 7890A-5975C, Agilent Technologies, USA). The analytical methods of heavy metals, water-soluble ions, PAHs, and VOCs were conducted through referral to previous work[1-5].

Results

Table S3. Concentrations of the main chemical compositions in human facial skin (mg/m^2).

Numerals	Acen	BkF	InP	Toluene	1,2-Dibromoethane	Chlorobenzene	p-Xylene	1,3,5-Trimethylbenzene	Ca	Cr	NO_3^-
1	0.0245	0.0004	0.0008	0.0040	0.0175	0.0026	0.0188	NA	1.3713	0.0049	0.1308
2	0.0107	0.0019	0.0331	0.0237	0.0245	0.0013	0.0125	NA	0.9212	0.0021	0.0105
3	0.0031	0.0020	0.0008	0.0034	0.0210	0.0017	0.0282	NA	0.5621	0.0047	0.0918
4	0.0059	0.0002	0.0038	0.0344	0.0035	0.0013	0.0157	0.0731	1.0672	0.0131	0.1906
5	0.0193	0.0059	0.0783	0.0320	0.0460	0.0034	0.0165	0.1350	0.9235	0.0128	0.2881
6	0.1006	0.0023	0.0548	0.0453	0.0230	0.0276	0.0055	0.2375	2.0801	0.0076	0.2500
7	0.0835	0.0020	0.0481	0.0119	0.1012	0.0045	0.0192	0.1479	0.9788	0.0268	0.2321
8	0.1733	0.0023	0.0495	0.0803	0.0874	0.0073	0.0398	0.1497	0.2730	0.0057	0.2273
9	0.0271	0.0009	0.0409	0.6292	0.0409	0.0040	0.0147	0.1871	0.0447	0.0094	0.1408
10	0.0468	0.0019	0.0109	0.0321	0.0736	0.0097	0.0354	0.0769	2.3178	0.0121	0.1513
11	0.0733	0.0015	0.0011	0.4653	0.0450	0.0015	0.0037	0.0606	1.9217	0.0172	0.2919
12	0.0923	0.0016	0.0639	0.0047	0.0695	0.0040	0.0220	NA	1.6265	0.0101	0.1992
13	0.1307	0.0009	0.0191	0.0720	0.0295	0.1508	0.0284	0.1876	0.7728	0.0227	0.1832
14	0.0999	0.0037	0.1177	0.0020	0.0590	0.0361	0.0059	0.0787	0.7768	0.0086	0.2323
15	0.1378	0.0038	0.0105	0.0061	0.0131	0.0012	0.0118	0.0285	1.1337	0.0104	0.1573
16	0.1335	0.0012	0.0009	0.0014	0.0720	0.0978	0.0137	0.1285	0.0521	0.0070	0.1973

NA means the concentration of composition lower than the detection limits.

References

1. Da, A., et al., Composition and origin of PM$_{2.5}$ in Mediterranean Countryside. *Environ. Pollut.* **2019**, 246, 294-302.
2. Li, et al., Investigation of PM$_{2.5}$ absorbed with heavy metal elements, source apportionment and their health impacts in residential houses in the North-east region of China. *Sustain. Cities Soc.* **2019**, 51(C) , 101690-101690.
3. Lin, Y.C., et al., Characterization and quantification of PM$_{2.5}$ emissions and PAHs concentration in PM$_{2.5}$ from the exhausts of diesel vehicles with various accumulated mileages. *Sci. Total Environ.* **2019**, 660(APR.10) , 188-198.
4. Niu, Y., et al., Source analysis of heavy metal elements of PM$_{2.5}$ in canteen in a university in winter. *Atmos. Environ.* **2021**, 244, 117879.
5. Sa'I, A., et al., Indoor generated PM$_{2.5}$ compositions and volatile organic compounds: Potential sources and health risk implications. *Chemosphere*. 2020, 255.
